# Effect of beneficial microbes applications on nutritional profiles of organic tomatoes revealed by LC‐MS‐qTOF metabolomics

**DOI:** 10.1002/jsfa.70316

**Published:** 2025-11-14

**Authors:** Daria Lotito, Alessia Staropoli, Maria Isabella Prigigallo, Giuseppina Iacomino, Claudio Gigliotti, Giovanni Bubici, Sergio Bolletti‐Censi, Matteo Lorito, Francesco Vinale

**Affiliations:** ^1^ Department of Veterinary Medicine and Animal Productions University of Naples Federico II Naples Italy; ^2^ Institute for Sustainable Plant Protection, National Research Council Portici Italy; ^3^ Institute for Sustainable Plant Protection, National Research Council Bari Italy; ^4^ Department of Agricultural Sciences University of Naples Federico II Portici Italy; ^5^ Cosvitec Società Consortile a Responsabilità Limitata Naples Italy

**Keywords:** antioxidants, metabolomics, *Streptomyces microflavus*, tomato berries, *Trichoderma afroharzianum*, *Trichoderma harzianum*

## Abstract

**BACKGROUND:**

The use of microorganisms and biostimulants is increasingly supported in agriculture because of their advantageous impact on plant disease management, growth enhancement and the synthesis of beneficial bioactive secondary metabolites (SMs). Tomato (*Solanum lycopersicum*) is an important crop and is consumed worldwide because it is an excellent source of natural compounds (i.e. beta‐carotene and flavonoids) and minerals useful for human health. Although the positive effects of individual microbial applications are well‐documented, the impact of microbial consortia is underexplored.

**RESULTS:**

In this study, the improvement of nutritional value of tomato (*S. lycopersicum* var. Heinz), by use of beneficial microorganisms, including selected strains of *Streptomyces microflavus* (S), *Trichoderma harzianum* (M10) and *Trichoderma afroharzianum* (T22), has been investigated. These microbes were applied on tomato plants, either as single inoculants or as microbial consortia. The effects were evaluated through statistical analysis of biological parameters. T22 treatments exhibited a significant increase in plant height (107.30 cm) compared to both control and M10‐based treatments (104.30 and 102.80 cm, respectively). The similarities observed in plant height between S‐treated plants (105.70 cm) and those treated with the combination of S and T22 (106.60 cm) highlight the potential beneficial effects of microbial consortia. Moreover, the berries were subjected to an untargeted metabolomic analysis by liquid chromatography‐mass spectrometry‐quadrupole‐time of flight that led to the identification of eighteen metabolites, including tomatine and its derivatives solafloridine. Multivariate analysis demonstrated differences in berries metabolic profiles, depending on the treatment applied. Specifically, T22‐based treatment increased the accumulation of most of the identified metabolites compared to untreated plants, whereas combined treatment S + T22 induced a major accumulation of solafloridine.

**CONCLUSION:**

Field microbial applications significantly induced the metabolic profile change of tomato and the accumulation of metabolites with nutraceutical value. © 2025 The Author(s). *Journal of the Science of Food and Agriculture* published by John Wiley & Sons Ltd on behalf of Society of Chemical Industry.

## INTRODUCTION

Tomato (*Solanum lycopersicum* L.) is widely recognized for its health‐promoting properties, attributed to its rich content of vitamins, minerals and bioactive compounds such as carotenoids, tocopherols and polyphenols (flavonoids, flavanones and flavones).^1^, ^2^, ^3^, ^4^, ^5^ Several epidemiological studies have shown that the consumption of tomato can aid in preventing chronic diseases, such as cancer and cardiovascular conditions,^6^, ^7^, ^8^, ^9^ largely as a result of compounds such as lycopene, a potent antioxidant that neutralizes freely available reactive oxygen species, in addition to reducing insulin‐like growth factor in blood and modulating the cellular pathways involved in cancer development.^10^, ^11^ Given the commercial importance of this crop in the human diet, research efforts are increasingly focused on improving the biochemical composition of the fruit, including the content of these potentially health‐beneficial components.

Despite the high demand for nutritionally dense tomatoes, cultivation faces challenges from both abiotic (i.e. drought, salinity and extreme temperatures) and biotic stressors (i.e. pests and pathogens).^12^, ^13^, ^14^ Abiotic stresses are major limiting factors to crop production globally, often leading to a substantial decline in yield, estimated to cause up to 50–70% of potential yield loss in major food crops.^14^ Furthermore, highly destructive biotic threats regularly compromise both yield and quality. These threats include insect pests, such as the whitefly (*Bemisia tabaci*) and the South American tomato pinworm (*Tuta absoluta*), which cause significant crop damage and major economic losses.^15^, ^16^, ^17^ Simultaneously, productivity is heavily impacted by numerous fungal (i.e. *Alternaria solani*, *Botrytis cinerea*, *Fusarium oxysporum* f. *sp. lycopersici* and *Phytophthora infestans*) and bacterial pathogens (i.e. *Pseudomonas syringae* pv. *tomato* and *Clavibacter michiganensis*).^18^ To mitigate the impact of these factors and to improve yield and nutritional quality, frequent applications of pesticides and synthetic fertilizers are necessary, although this presents threats to ecological, environmental, and animal and human safety.^19^ Among the various strategies aimed at reducing the risks associated with chemical applications and dependency on their use, a global shift towards sustainable agriculture strategies is making the use of microbial biostimulants and biological control agents (and their bioactive metabolites) one of the most promising approaches for substituting synthetic agro‐chemicals.^18^, ^20^, ^21^, ^22^ Biostimulants are substances or microorganisms applied to plants with the aim of stimulating natural processes, thereby improving nutrient use efficiency, abiotic stress tolerance and crop quality traits.^23^, ^24^ The use of biostimulants has increased significantly as a result of their beneficial effects on plants, which include the stimulation of primary and secondary metabolism to boost yield and promote the accumulation of bioactive compounds.^25^

*Trichoderma*‐based products are globally marketed for protecting crops against diverse plant pathogens and enhancing plant growth and productivity through various mechanisms of action.^26^, ^27^ *Streptomyces* is recognized as one of the major sources of bioactive natural compounds used for pharmaceutical and agricultural applications.^28^, ^29^ Moreover, various species of *Streptomyces* synthesize numerous hydrolytic enzymes that aid the producing strain during the interactions and competition with other microorganisms. These characteristics underscore streptomycetes as highly effective biological control agents.^28^, ^29^, ^30^

In the present study, *Trichoderma harzianum* strain M10, *Trichoderma afroharzianum* strain T22 and one strain of *Streptomyces microflavus* (AtB‐42) were selected for their efficacy against phytopathogen agents and pests, as well as for plant growth promoting activity.^31^, ^32^, ^33^, ^34^, ^35^ These microbes were applied individually and in combination to tomato plants because recent research has shown the benefits of using microbial consortia or combined treatments.^36^, ^37^, ^38^ Although a growing body of literature confirms that microbial inoculations can enhance single quality traits in crops, comprehensive studies linking microbial application to the global metabolomic profile of tomato fruit remain limited, particularly considering the combined application of different microbial genera, such as *Trichoderma* and *Streptomyces*. The objective of this study was to assess the impact of these microbial treatments on the growth of tomato plants and metabolomic profiles of tomato berries, by an untargeted and pseudo‐targeted metabolomic approach, taking advantage of mass spectrometry methodologies. This approach allowed for a comprehensive analysis of the changes in metabolites induced by microbial inoculation, providing insights into how these treatments influence tomato berry development and biochemical composition.

## MATERIALS AND METHODS

### Chemicals and reagents

Liquid chromatography‐mass spectrometry (LC‐MS) grade solvents (acetonitrile, water, methanol and formic acid) and reference standards of tomatidine, naringenin and rutin were purchased from Sigma‐Aldrich (St Louis, MO, USA). Stock solutions of reference standards were obtained by suspension in methanol at a concentration of 1 mg mL^–1^. Substrates used to cultivate microbial strains [potato dextrose agar (PDA) and tryptone soy agar (TSA)] were purchased from HI‐MEDIA (Pvt Ltd, Mumbai, India). Petri dishes and microtubes used for microbial cultivation and for metabolites extraction were purchased from Thermo Fisher Scientific (Waltham, MA, USA).

### Microbial strains

The three strains, *T. afroharzianum* T22, *T. harzianum* M10 and *S. microflavus* AtB‐42, were selected for their documented, complementary biocontrol and plant growth‐promoting traits,^34^, ^35^, ^36^, ^37^ and were prepared as follows. *Trichoderma afroharzianum* strain T22 was isolated from the commercial product Trianum‐P (Koppert Biological Systems, Rotterdam, The Netherlands). The fungus was cultured on PDA and incubated at 25 °C until it reached complete sporulation. Conidia were collected with sterile distilled water by scraping the surface of the culture. The concentration of conidia was adjusted to achieve the desired concentration for the inoculum.

*Trichoderma harzianum* strain M10 was obtained from the collection available at the Department of Agricultural Sciences of the University of Naples Federico II (Naples, Italy) and cultured as reported for the T22 strain.

*Streptomyces microflavus* AtB‐42 was obtained from the collection available at the Institute for Sustainable Plant Protection (Bari, Apulia, Italy) and cultured on TSA. Inoculum was prepared according to Staropoli *et al*. (2021).^37^ Spore/conidia concentration of M10 and AtB‐42 was estimated with a Thoma counting chamber and adjusted to the desired value for the inoculum with sterile distilled water (10^7^ spores/mL^–1^).

Compatibility between *S. microflavus* AtB‐42 and *T. harzianum* M10 was previously investigated by Prigigallo *et al*. (2023),^36^ and preliminary *in vitro* compatibility tests were conducted for the *S. microflavus* AtB‐42 and *T. afroharzianum* T22 combination prior to their use in the field experiment.

### Field trial

The trial was established in an open field located at Pietramelara, Campania, Italy. In June, 40‐day‐old tomato seedlings (*S. lycopersicum* var. Heinz5108) were transplanted in single rows at 10‐cm distance on the row and 20 cm between rows. The trial consisted of six treatments, control (Ctrl), *T. afroharzianum* T22 (T22), *S. microflavus* AtB‐42 (S), *T. harzianum* M10 (M10) and two mixes (S_T22 and S_M10). All the treatments were carried out three times: at transplanting by dipping roots for 15 min in conidia or spore suspensions (1 × 10^7^ conidia/spores mL^–1^), 1 and 2 months later by soil drenching 25 mL per plant of conidia or spore suspensions (1 × 10^7^ conidia/spores mL^–1^).

A randomized complete block design with two blocks was adopted. Blocks were separated by untreated plants (extra plants) to avoid any contamination through the applications of microorganisms. Eighty plants per treatment in each block were used (see Supporting information, Fig. S1). The experiment was repeated twice.

Plants were cultivated according to the agronomic organic practices commonly used in the farm. At the end of crop cycle (four months), 10 plants per treatment (five from each block) were harvested and biometric parameters were measured (plant height, root length from primary root). Tomato yield was determined by the number of fruits per plant and the average fruit weight recorded from a representative subsample of plants within each plot. Mean yield values were computed for each treatment, and differences among treatments were analyzed by analysis of variance (ANOVA). Ten biological replicates per treatment (five from each block) of tomato berries were frozen in liquid nitrogen immediately after harvesting and then stored at −80 °C for further metabolomic analysis. Microbial colonization was verified at the end of the experiment by the serial dilution and plating technique, and their abundance was assessed through the counting of colony‐forming units (CFUs) per gram of soil.

### Metabolites extraction

Tomato berries were freeze‐dried under vacuum and powdered using a homogenizer (T25 digital ULTRA‐TURRAX®; IKA‐Werke GmbH & Co. KG, Staufen, Germany) prior to the extraction in organic solvent. Specifically, 100 mg of powder for each sample were suspended in 2 mL of methanol (MeOH), thoroughly vortexed, sonicated for 5 min in an ultrasonic bath (Sonorex, Bandelin electronic GmbH & Co. K, Berlin, Germany) and stored for 1 h at 4 °C. Samples were then centrifuged at 17 005 x g for 10 min at 4 °C. Each supernatant was recovered and a 500‐μL aliquot was diluted 1:10 in MeOH and analyzed using a LC‐MS quadrupole‐time of flight (qTOF) system.

### LC‐MS analysis

Untargeted metabolomics analyses were performed using an Agilent HP 1260 Infinity Series liquid chromatograph coupled with a qTOF mass spectrometer and equipped with a diode array detector system (Agilent Technologies, Santa Clara, CA, USA). An Adamas® C‐18 column (4.6 × 50 mm, 3.5 μm; SepaChrom Srl, Rho, Mi, Italy), held at a constant temperature of 25°C, was used for chromatographic separation. The analyses were carried out following a previously described method.^37^

### Statistical analysis

Biometric parameters data were statistically analyzed (one‐way ANOVA) with Minitab statistical software (Minitab, LLC, State College, PA, USA). The assumptions of normality and homoscedasticity were verified for all data sets prior to analysis. Normality was assessed using the Shapiro–Wilk test (*P* > 0.05) and homogeneity of variances was assessed with the Levene's test (*P* > 0.05). Multiple comparison of means was performed by the least significant difference test with a 0.05 significance level.

Statistical analysis of metabolomics data was performed using Mass Profile Professional, version 13.1.1 (MPP) (Agilent Technologies) and MetaboAnalyst, version 6.0^39^ (https://www.metaboanalyst.ca). MPP was used for alignment, normalization of raw data and molecular features identification, obtained by comparison of the monoisotopic mass with data present in an in‐house built plant database and with data available in the literature. Among the detected molecules, only those with a mass error below 5 ppm and a sufficient score (over 70) were reported. Reference standard solutions were used to confirm the identification of tomatidine, naringenin and rutin. Identified metabolites were then classified using the ClassyFire web‐based tool^40^ (https://cfb.fiehnlab.ucdavis.edu). All of the molecular features (unidentified and identified) were then grouped by the treatment applied in the field (i.e. single strains or microbial consortium) and these groups were initially subjected to multivariate analyses [principal component analysis (PCA) and partial least square‐discriminant analysis (PLS‐DA)]. PCA was conducted to look for trends between and within conditions; PLS‐DA was performed to identify molecular features responsible for the separation between groups, by computation of variable importance in projection (VIP).^41^ Prior to interpretation, the robustness and validity of the PLS‐DA models for both positive and negative ionization modes were rigorously assessed through cross‐validation and permutation testing. The optimal number of latent variables (components) for the PLS‐DA model was determined by evaluating predictive power using five‐fold cross‐validation with a maximum search limit of five components. The predictive ability was quantified using *Q* ^2^ statistics. Model validity was further confirmed via a permutation test performed with 100 random iterations. Differentially accumulated metabolites were then screened by ANOVA and VIP (*P* < 0.05 and VIP > 1.0). Significantly different metabolites were also subjected to hierarchical clustering analysis (HCA) and fold change analysis with a cut‐off = 2, using MetaboAnalyst version 6.0.

## RESULTS AND DISCUSSION

### Plant growth

Integrating microorganisms and biostimulants into agricultural practices can lead to healthier crops, improved soil fertility, reduced chemical inputs and, ultimately, more sustainable and productive farming systems.^42^ In the present study, we investigated the effects of single and combined application of S. *microflavus* strain AtB‐42 (S), *T. harzianum* strain M10 (M10) and *T. afroharzianum* T22 (T22) on the growth of tomato plants in a field environment, simultaneously evaluating alterations in the metabolic profile of the berries upon treatments. The effects were evaluated through statistical analysis of biological parameters (Table 1). T22 treatments demonstrated a significant increase in plant height (107.30 cm) compared to both control and M10‐based treatments (104.30 and 102.80 cm, respectively), as also reported by recent studies on other crops.^42^, ^43^, ^44^ Moreover, the similarities observed in plant height between S‐treated plants (105.70 cm) and those treated with the combination of S and T22 (S_T22, 106.60 cm) suggest that no negative interaction occurred in the consortium with respect to plant height because the final performance remained equivalent to the single treatments. Although specific interactions between *Trichoderma* and *Streptomyces* strains need further investigation, a recent study by Prigigallo *et al*.^36^ indicated the potential for cooperative interactions between these microbial genera. In terms of root parameters, a similar trend among T22, S alone and the combination S_T22 can be noted (24.10, 23.30 and 24.50 cm, respectively). Moreover, the substantial differences observed between plants treated with M10 and T22‐based treatments further highlight the specificity of microbial effects on root development. Although M10‐treated plants exhibited inferior root growth (20.70 cm) compared to T22‐treated plants, the similarity between S‐treated plants and the combination of S and M10 (S_M10, 22.40 cm) suggests a moderate influence of *Streptomyces* strains on root length. These findings are consistent with recent research by Rahman *et al*.^45^ and Iacomino *et al*.,^46^ highlighting the variable effects of different microbial strains on root architecture in tomato plants. Among all treatments, only T22 yield was significantly higher (10% higher) compared to control (data not shown). No natural plant pathogen attack has been recorded during the experiments.

**Table 1:** Effect on the biometric parameters of tomato plants treated with: *Trichoderma afroharzianum* T22 (T22); *Streptomyces microflavus* AtB‐42 (S); *Trichoderma harzianum* M10 (M10); two microbial combinations [*S. microflavus* + *T. afroharzianum* (S_T22) and *S. microflavus* + *T. harzianum* (S_M10)] and a water control (Ctrl)

Treatment	Plant height (cm)	Root length (cm)
Ctrl	104.30 ± 1.70 bc	22.60 ± 2.88 bc
T22	107.30 ± 3.50 a	24.10 ± 2.13 a
M10	102.80 ± 1.31 c	20.70 ± 1.33 c
S	105.70 ± 1.33 ab	23.30 ± 1.76 ab
S_T22	106.60 ± 2.10 a	24.50 ± 2.12 a
S_M10	102.70 ± 1.54 c	22.40 ± 1.35 ab
*N*	10	10
Significance	***	**

*N* = number of replicates; **P ≤ 0.001 and ***0.0001, respectively.

Data are presented as the mean ± SD. Different lowercase letters within each column indicate a significant difference differences according to a least significance difference test (*P* < 0.05).

Microbial colonization was verified at the end of the experiment. Both *Trichoderma* strains and *Streptomyces microflavus* AtB‐42 were found in treated soil at 10^5^ cfu g^–1^ and 10^6^ cfu g^–1^, respectively (data not shown).

### Untargeted metabolite profiling

The untargeted metabolomic analysis of tomato berries led to the detection of 189 molecular features (100 in positive and 89 in negative ionization mode), of which 39 were putatively identified by comparison of monoisotopic masses and mass spectra (Table 2).

**Table 2:** Putatively identified metabolites in tomato berries extracts analyzed by LC‐MS‐qTOF

Adducts	RT (min)	Compound	Formula	Ion *m/z* (Da)
M‐Na^+^/M‐H^+^	0.890	l‐Homoserine	C_4_H_9_NO_3_	124.0381/118.0514
M‐H^+^	0.896	l‐Asparagine	C_4_H_8_N_2_O_3_	131.0468
M‐H^+^	0.897	l‐Glutamine	C_5_H_10_N_2_O_3_	145.0625
M‐H^+^	0.909	Linamarin	C_10_H_17_NO_6_	248.1126
M‐H^+^	0.916	l‐Glutamic acid	C_5_H_9_NO_4_	130.0495
M‐H^+^	0.930	Sucrose	C_12_H_22_O_11_	381.0792
M‐H^+^	0.936	Trigonelline	C_7_H_7_NO_2_	176.0105
M‐H^+^	0.939	Gluconic acid	C_6_H_12_O_7_	195.0517
M‐H^+^	0.961	Quinic acid	C_7_H_12_O_6_	191.057
M‐H^+^	0.970	Adenosine	C_10_H_13_N_5_O_4_	268.1038
M‐H^+^	0.970	Galactonic acid	C_6_H_12_O_7_	195.0517
M‐H^+^	0.975	l‐Tyrosine	C_9_H_11_NO_3_	180.0673
M‐H^+^	0.998	Adenine	C_5_H_5_N_5_	136.0621
M‐H^+^	1.530	l‐Phenylalanine	C_9_H_11_NO_2_	166.086
M‐H^+^	1.531	Tyramine	C_8_H_11_NO	120.0808
M‐H^+^	1.538	Benzeneacetaldehyde	C_8_H_8_O	103.054
M‐H^+^	3.440	3‐Indolyllactic acid	C_11_H_11_NO_3_	188.0707
M‐H^+^	3.663	1‐Caffeoyl‐β‐d‐glucose	C_15_H_18_O_9_	341.0877
M‐H^+^	3.690	Tryptophol	C_10_H_11_NO	144.0808
M‐H^+^	3.811	5‐Caffeoylquinic acid	C_16_H_18_O_9_	353.0871
M‐H^+^	3.983	Apiorutin	C_32_H_38_O_20_	741.1883
M‐H^+^	4.024	Lycoperoside F	C_58_H_95_NO_29_	1270.6066
M‐H^+^	4.129	Kaempferol 3,7‐di‐*O*‐glucoside	C_27_H_30_O_16_	611.1613
M‐H^+^	4.132	Delphin	C_27_H_31_O_17_	609.1464
M‐H^+^	4.165	Rutin	C_27_H_30_O_16_	611.1611
M‐H^+^	4.165	Quercetin	C_15_H_10_O_7_	303.0499
M‐H^+^	4.349	23R‐Acetoxytomatine	C_52_H_85_NO_23_	1092.559
M‐H^+^	4.374	Tomatine	C_50_H_83_NO_21_	1034.5548
M‐H^+^	4.375	Tomatidine galactoside	C_33_H_5_NO_7_	578.4058
M‐H^+^	4.375	Tomatidine	C_27_H_45_NO_2_	416.3525
M‐H^+^	4.381	Solafloridine	C_27_H_45_NO_2_	416.3525
M‐H^+^	4.630	Prunin	C_21_ H_22_ O_10_	433.1138
M‐H^+^	4.786	*trans*‐*p*‐Ferulyl alcohol 4‐*O*‐[6‐(2‐methyl‐3‐hydroxypropionyl)] glucopyranoside	C_20_H_28_O_10_	427.1608
M‐H^+^	5.057	Cyanidin	C_15_H_11_O_6_	287.0562
M‐H^+^	5.059	(+)‐Gallocatechin	C_15_H_14_O_7_	287.0562
M‐H^+^	5.308	Butin	C_15_H_12_O_5_	273.0761
M‐H^+^	5.312	Naringenin	C_15_H_14_O_6_	273.0759
M‐H^+^	5.431	Peonidin	C_16_H_13_O_6_	301.0708
M‐H^+^	6.215	(−)‐Phytuberin	C_17_H_26_O_4_	293.17

RT, retention time; m/z, mass‐to‐charge ratio. Naringenin, rutin and tomatidine identification was confirmed with reference standards. Each metabolite is reported together with retention time, formula and ion monoisotopic masses of the detected adducts (either positive or negative ionization mode).

**Figure 1:**
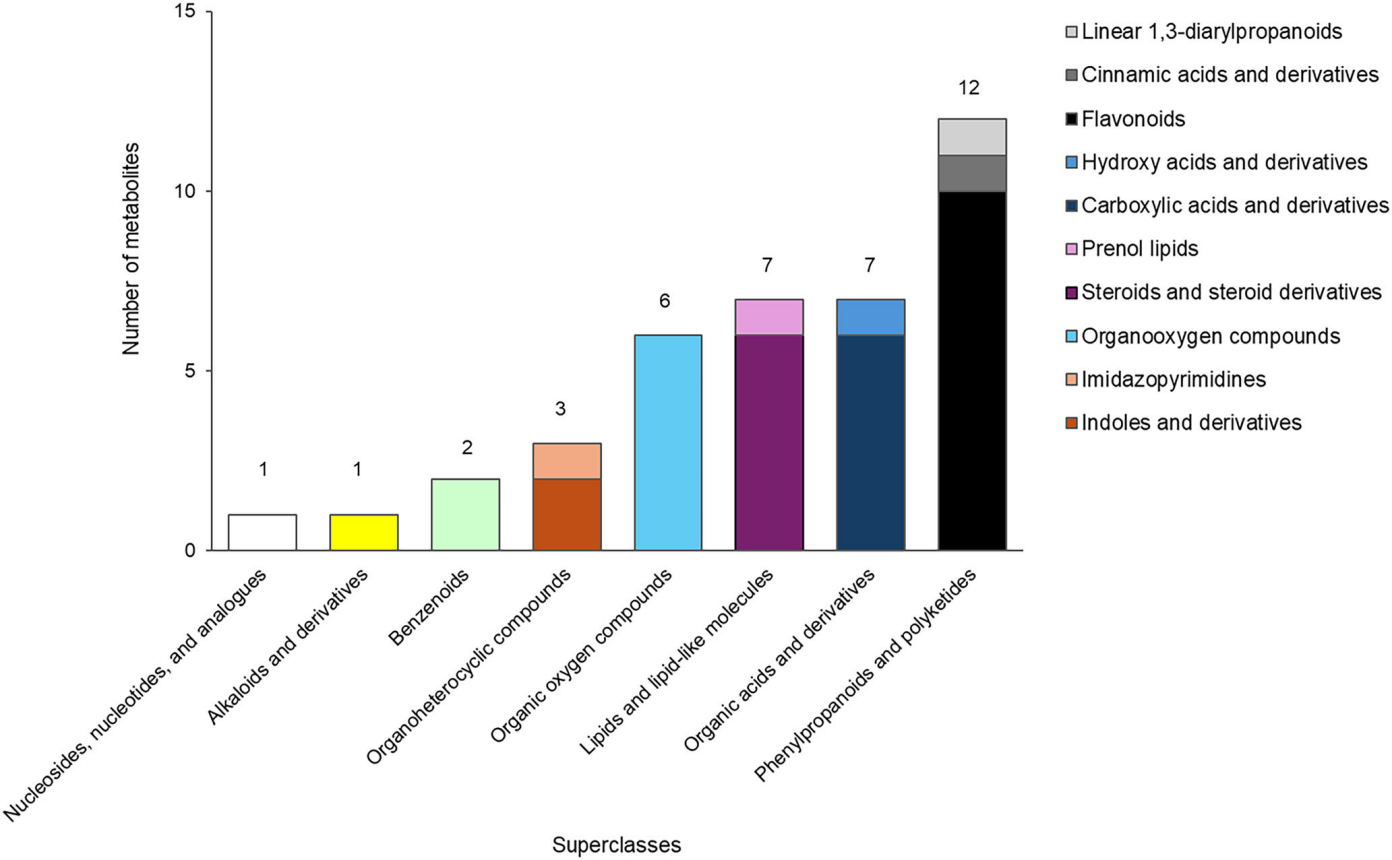
Classification of identified metabolites [electrospray ionization in positive (ESI^+^) and negative mode (ESI^−^)] of tomato berries extracts. The *x*‐axis represents the metabolite groups at the superclass level; the *y*‐axis represents the number of metabolites for each superclass.

Metabolite classification showed that identified metabolites mainly belong to phenylpropanoids and polyketides, organic acids, organic oxygen and derivatives, and lipid‐like molecules, at the superclass level. Flavonoids were the major molecules at the class level, followed by carboxylic acids, steroids and derivatives (Fig. 1).

**Figure 2:**
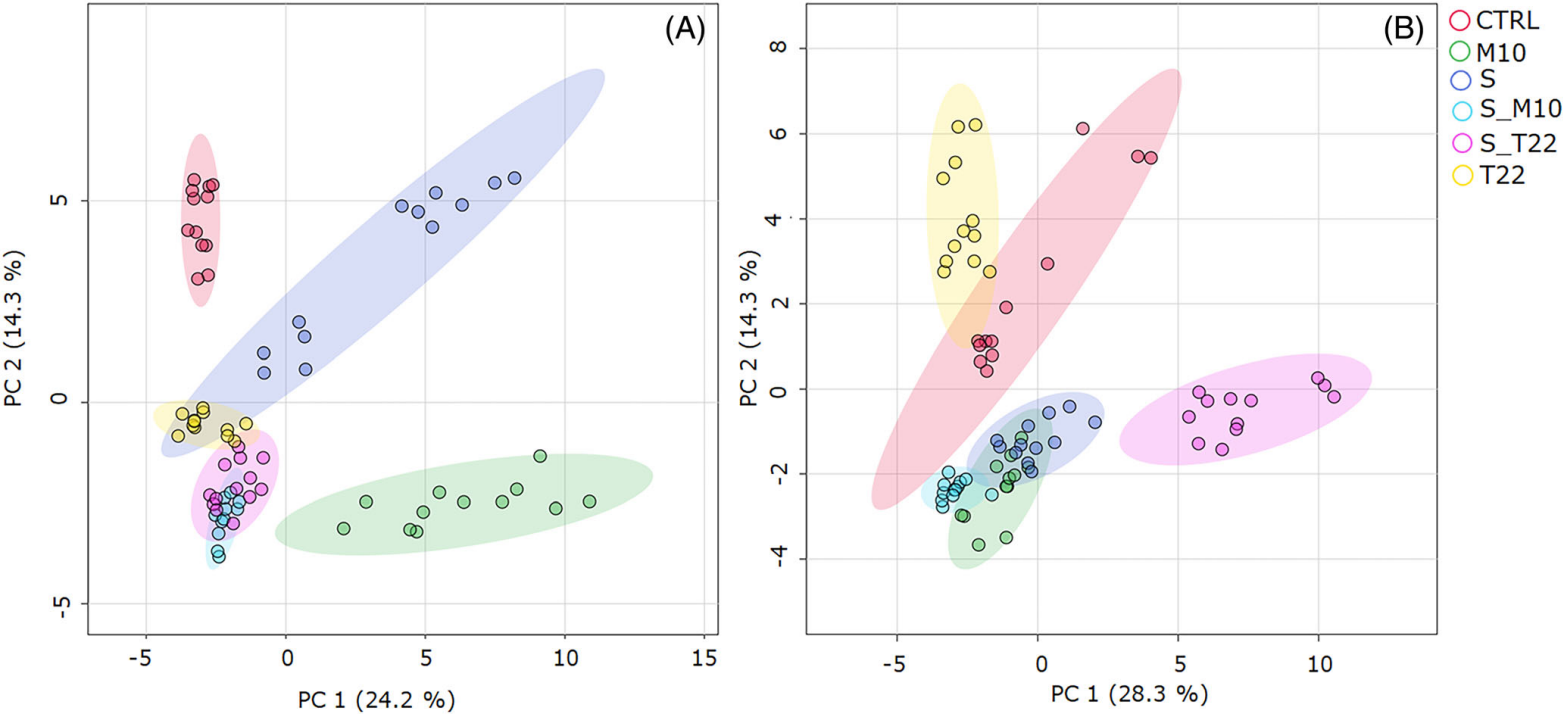
PCA of tomato berries metabolomic profile. Plants were treated with single strain (*T. harzianum* M10, *T. afroharzianum* T22 and *S. microflavus* AtB‐42) or microbial consortia inoculants (S_M10 and S_T22). Data were obtained by LC‐MS‐qTOF analysis. (A) PCA scores plot in electrospray ionization in positive mode (ESI^+^). (B) PCA scores plot in electrospray ionization in negative mode (ESI^−^). Each treatment is depicted in a different color: CTRL in red; M10 in green; T22 in yellow; S in violet; S_M10 in light blue; S_T22 in pink.

Aligned and normalized raw data were then used to perform a PCA aiming to obtain a global view of the metabolomic profiles and to identify trends among and within sample groups of tomato berries for which plants were subjected to different treatments. Multivariate analysis revealed a distinction among the six groups of samples that cluster separately in both ionization modes with few differences (Fig. 2). As for positive mode (Fig. 2(A)), there was a separation along both components of four groups representing control (in red), M10‐based treatment (in green), combined treatments that cluster together (S_M10 in light blue and S_T22 in pink) and single strain treatments (T22 and S, in yellow and violet, respectively), which are closer in the components space. In Fig. 2(B), the control group is closer to T22 along component 1 and these two groups are separated along component 2 from the other four; M10, S and S_M10 groups are closer, whereas the combined treatment S_T22 group is far way from both components. Moreover, each multivariate analysis showed an unsupervised separation within the groups, particularly evident in S and M10 groups in positive ionization mode, and C, T22 and M10 groups in negative ionization mode (Fig. 2). This spread reflected the biological and environmental heterogeneity of the field experiment.

**Figure 3:**
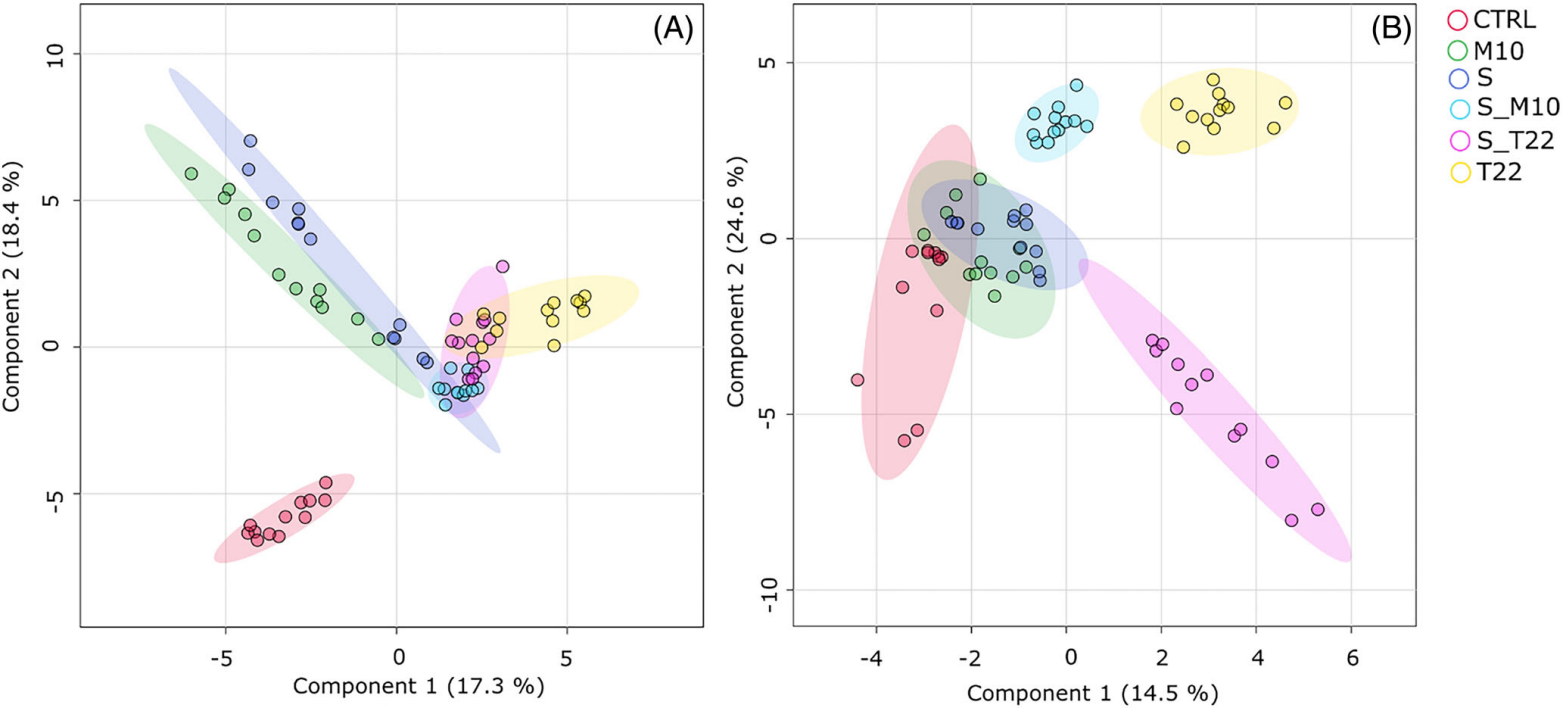
PLS‐DA of tomato berries metabolomic profile Plants were treated with single strain (*T. harzianum* M10, *T. afroharzianum* T22 and *S. microflavus* AtB‐42) or microbial consortia inoculants (S_M10 and S_T22). Data were obtained by LC‐MS‐qTOF analysis. (A) PLS‐DA scores plot in electrospray ionization in positive mode (ESI^+^). (B) PLS‐DA scores plot in electrospray ionization in negative mode (ESI^−^). Each treatment is depicted in a different color: CTRL in red; M10 in green; T22 in yellow; S in violet; S_M10 in light blue; S_T22 in pink.

Because of the interesting differences revealed by the unsupervised analysis, metabolomics data were further processed. Specifically, PLS‐DA was conducted to highlight difference between the groups and to screen the metabolites more affected by the treatments (Fig. 3). By contrast to PCA, PLS‐DA provided a more robust method for supervised classification. PLS‐DA not only aided noise reduction but also allowed for the extraction of valuable information.^47^ By emphasizing the systematic variation relevant to classification while minimizing orthogonal variation, PLS‐DA enhanced the differentiation between classes and improved the interpretability of the model. PLS‐DA score plots, obtained for both ionization modes, reinforced the insight obtained from the PCA because the separation between sample groups became even more pronounced, especially for negative ionization mode (Fig. 3(B)). Additionally, the reduced spread of individual samples within each group demonstrated the repeatability of the metabolomics method.

The robustness and predictive ability of the PLS‐DA models were validated through cross‐validation and permutation testing (see Supporting information, Figs S2 and S3). The five‐fold cross‐validation confirmed excellent predictive power, yielding a *Q* ^2^ of 0.95 for the positive mode and 0.94 for the negative mode using five components, demonstrating high predictive stability. Furthermore, the permutation tests (100 iterations) demonstrated that the observed class separation was highly significant, resulting in an empirical *P* < 0.01 (0/100 permutations) for both modes, ultimately ruling out the possibility of overfitting or chance separation.

### Pseudo‐targeted metabolomics

**Figure 4:**
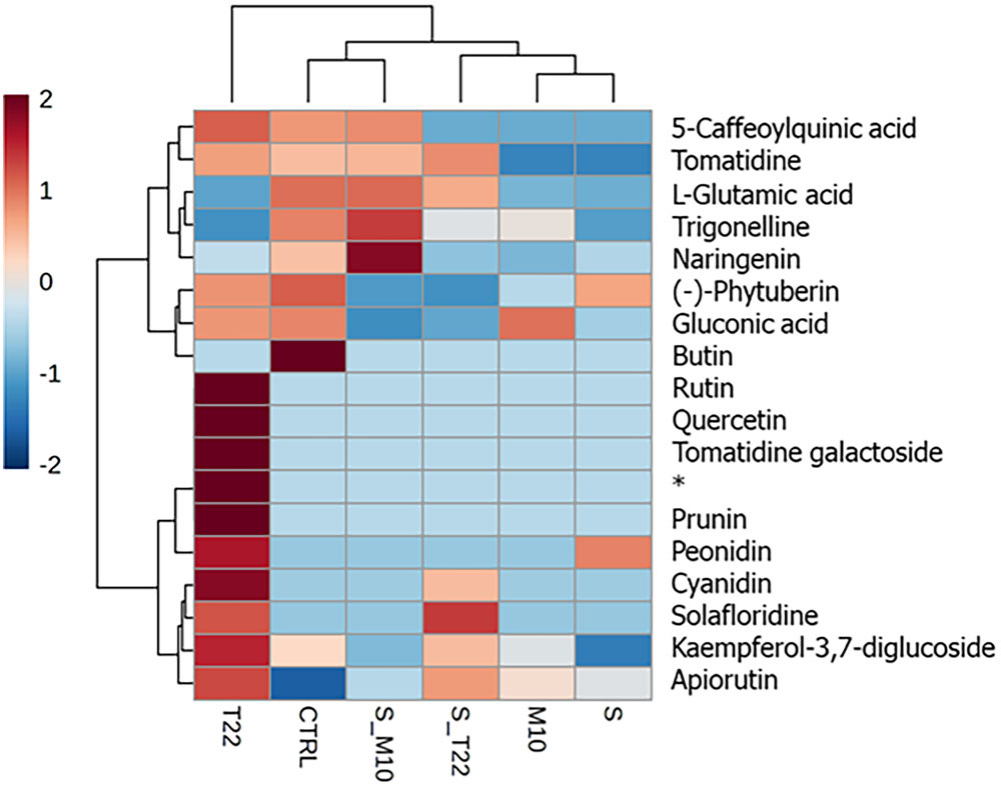
Heat map and dendrogram obtained by comparison of metabolomics profiles of tomato berries. Plants were treated with single strain (*T. harzianum* M10, *T. afroharzianum* T22 and *S. microflavus* AtB‐42) or microbial consortia inoculants (S_M10 and S_T22). Data were obtained by LC‐MS‐qTOF analysis. The range of colors refers to normalized intensity values, ranging from blue (less abundant) to red (most abundant). Reported metabolites are significant among treatments (*P* < 0.05 and VIP > 1.0). *trans‐*p*‐Ferulylalcohol 4‐*O*‐[6‐(2‐methyl‐3‐hydroxypropionyl)] glucopyranoside.

To identify the metabolites that contributed the most to the observed differences among treatments, differentially accumulated metabolites were screened based on their VIP values (obtained from PLS‐DA; see also Supporting information, Fig. S2 and Table S1) and univariate statistical analysis (ANOVA). Metabolites with VIP values >1.0 and *P* < 0.05 were considered significant (see Supporting information, Fig. S4 and Table S2). The 18 metabolites resulted from the screening (Table 3) were also subjected to HCA to easily visualize the differences of abundances among treatments (Fig. 4).

**Table 3:** Differentially accumulated metabolites of tomato berries, obtained by PLS‐DA and analysis of variance (ANOVA). Data were obtained by LC‐MS‐qTOF analysis in both positive and negative ionization mode

Compound	*P*	VIP
Naringenin	3.57 × 10^–27^	2.2395
Butin	3.57 × 10^–27^	2.1381
5‐Caffeoylquinic acid	7.99 × 10^–18^	1.9712
*trans*‐*p*‐Ferulylalcohol 4‐*O*‐[6‐(2‐methyl‐3‐hydroxypropionyl)] glucopyranoside	7.09 × 10^–18^	1.9608
Prunin	1.05 × 10^–14^	1.7361
Solafloridine	1.81 × 10^–10^	1.7099
l‐Glutamic acid	1.89 × 10^–7^	1.6547
Cyanidin	7.08 × 10^–12^	1.5423
Tomatidine galactoside	1.33 × 10^–26^	1.5278
Apiorutin	1.55 × 10^–30^	1.5224
Rutin	9.38 × 10^–32^	1.437
Trigonelline	8.15 × 10^–9^	1.3888
Quercetin	1.81 × 10^–22^	1.347
(−)‐Phytuberin	3.71 × 10^–9^	1.1742
Gluconic acid	2.49 × 10^–5^	1.0498
Peonidin	7.94 × 10^–17^	1.0361
Kaempferol 3,7‐di‐*O*‐glucoside	1.31 × 10^–25^	1.0342
Tomatidine	4.79 × 10^–8^	1.0044

The HCA heatmap revealed distinct patterns in metabolite abundance across different microbial treatments, indicating significant variations in metabolic profiles. These differences highlighted the impact of specific treatments on the tomato metabolic pathways, which can affect both the plant's resilience and the nutritional and health benefits for consumers. Similarly, Mhlongo *et al*.^48^ and Coppola *et al*.^49^ observed a change in the metabolic profile of tomato plants upon microbial‐based treatments. Various classes of metabolites were modulated and thus further studied to evaluate the effects of treatments in terms of relative abundance variations (Fig. 4). Phenolics, the main bioactive compounds of tomato, are a class of secondary metabolites with a huge structural diversity, varying from simple phenolic acids to more complex polyphenols such as flavonoids. They are considered health‐promoting substances as a result of antioxidant, anti‐inflammatory and anti‐mutagenic properties.^6^ Rutin, apiorutin, quercetin, prunin, peonidin, cyanidin and kaempferol‐3,7‐diglucoside exhibited the greater abundance increase upon T22‐based treatment, ranging from 7 to 26 times higher compared to control group (see Supporting information, Table S3), whereas naringenin content was affected by the combination of *S. microflavus* and M10 (24 and 15 times higher compared to M10 and S‐based treatments, respectively, and two times higher compared to control). Tareq *et al*.^50^ focused on the impact of salinity on the metabolite profiles of tomato cultivars, revealing significant variations in flavonoids and other phenolic compounds, suggesting that both biotic and abiotic factors can modulate similar metabolic pathways, potentially enhancing stress tolerance and nutritional quality.

Alkaloids are another represented class of metabolites in the Solanaceae family, particularly in tomatoes. These molecules play an important role in the plant resistance against pathogens, such as fungi, bacteria, viruses and insects, and are also recognized for their anti‐inflammatory, antiviral, anticancer activities.^51^, ^52^, ^53^ Among glycoalkaloids, tomatidine, its glycosylated form, trigonelline and solafloridine showed variable levels across treatments; specifically, T22 induced a greater accumulation of tomatidine (two times higher compared to control group) and its glycosylated form (10 times higher compared to control), as also reported by Staropoli *et al*.^54^ Lower levels were found in S‐ and M10‐treated plants, suggesting that the metabolism of these molecules is dependent on different microbial actions. Li *et al*.^55^ observed how microbial action and moist‐heat treatments impact the non‐volatile metabolite profile of Pu‐Erh tea, significantly altering the abundance of various metabolites, including amino acids, phenolic compounds and alkaloids. This is similar to the changes observed in tomato berries subjected to different microbial treatments, where distinct metabolic shifts were noted, especially in flavonoids and glycoalkaloids.

In conclusion, the use of microbial biostimulants in agriculture has shown promising results in enhancing plant growth and nutrient uptake, at the same time as influencing the synthesis of beneficial bioactive secondary metabolites. The present study specifically evaluated the effects of *S. microflavus* strain AtB‐42, *T. harzianum* strain M10 and *T. afroharzianum* T22 on the growth and metabolic profile of tomato plants. The results indicated significant improvements in plant height and root development with T22‐based treatments. Metabolomic analysis revealed substantial changes in the metabolic profiles of tomato berries, particularly in the abundance of polyphenols and glycoalkaloids, which are crucial for plant defense and human health. These findings highlight the potential of microbial consortia in agriculture, underscoring the need for further research into the specific interactions between microbial strains to fully harness their benefits.

## FUNDING

This work was supported by MISE (grant number Protection F/050421/01‐03/X32). This study was carried out within the Agritech National Research Center and received funding from the European Union Next‐Generation EU (PIANO NAZIONALE DI RIPRESA E RESILIENZA (PNRR) – MISSIONE 4 COMPONENTE 2, INVESTIMENTO 1.4 – D.D. 1032 17/06/2022, CN00000022). This manuscript reflects only the views and opinions of the authors and neither the European Union nor the European Commission can be considered responsible for them.

## CONFLICTS OF INTEREST

The authors declare that they have no conflicts of interest.

## AUTHOR CONTRIBUTIONS

FV, ML and GB conceived the study. FV and SBC provided resources. MIP and DL carried out microbial preparations. CG conducted the field trial. AS, G. and DL performed LC‐MS analysis. AS curated the data. AS and DL wrote the original draft. AS, GI, FV and GB reviewed and edited the manuscript. All authors have approved the final version of the manuscript submitted for publication.

## Supporting information


**Figure S1.** Layout of the experimental design. Tomato plants were treated with *T. harzianum* M10 (M10), *T. afroharzianum* T22 (T22), *Streptomyces microflavus* AtB‐42 (S) and two microbial consortia (S_M10 and S_T22). CTRL refers to untreated plants.
**Figure S2.** Validation of the PLS‐DA model (positive ionization mode dataset). Left: Cross‐validation plot (5‐fold CV): performance metrics for the PLS‐DA model assessed across one to five components. The plot illustrates the model's goodness of fit (*R* ^2^), predictive ability (*Q* ^2^) and classification accuracy. Right: Permutation test (100 iterations): histogram illustrating the distribution of test statistics from 100 random permutations of the class labels (null distribution).
**Figure S3.** Validation of the PLS‐DA model (negative ionization mode dataset). Left: Cross‐validation plot (5‐Fold CV): performance metrics for the PLS‐DA model assessed across one to five components. The plot illustrates the model's goodness of fit (*R* ^2^), predictive ability (*Q* ^2^) and classification accuracy. Right: Permutation test (100 iterations): histogram illustrating the distribution of test statistics from 100 random permutations of the class labels (null distribution).
**Figure S4.** Important features (unidentified and identified) of tomato berries extracts, identified by partial least square discriminant analysis. Plants were treated with single strain or microbial consortia inoculants (*T. harzianum* M10, *T. afroharzianum* T22 and *S. microflavus* AtB‐42). Data were obtained by LC‐MS‐qTOF analysis. The first 15 features are reported from the highest to the lowest VIP value. The colored boxes on the right indicate the relative abundances of the corresponding metabolite in each group. Left: PLS‐DA scores plot in electrospray ionization in positive mode (ESI^+^). Right: PLS‐DA scores plot in electrospray ionization in positive mode (ESI^−^).
**Table S1.** Important features (unidentified and identified) of tomato berries extracts, obtained by partial least square discriminant analysis. Plants were treated with single strain or microbial consortia inoculants (*T. harzianum* M10, *T. afroharzianum* T22 and *S. microflavus* AtB‐42). Data were obtained by LC‐MS‐qTOF analysis with electrospray ionization in positive (ESI^+^) and negative (ESI^−^) mode. Variable importance in projection values are calculated for each component of PLS‐DA.
**Table S2.** Significantly different molecular features (unidentified and identified) of tomato berries extracts, obtained by analysis of variance (ANOVA, *P* < 0.05). Plants were treated with single strain or microbial consortia inoculants (*T. harzianum* M10, *T. afroharzianum* T22 and *S. microflavus* AtB‐42). Data were obtained by LC‐MS‐qTOF analysis with electrospray ionization in positive (ESI^+^) and negative (ESI^−^) mode.
**Table S3.** Absolute fold change values for a selected group of differentially accumulated metabolites in tomato berries. Metabolites were initially selected based on partial least squares‐discriminant analysis (PLS‐DA) and analysis of variance (ANOVA). Fold change values >1 indicate up‐regulation (enrichment) and values <1 indicate down‐regulation (depletion). Data were obtained by LC‐MS‐qTOF analysis in both positive and negative ionization mode. Plants were treated with single strain or microbial consortia inoculants (*T. harzianum* M10, *T. afroharzianum* T22 and *S. microflavus* AtB‐42).

## Data Availability

The data that support the findings of this study are available from the corresponding author upon reasonable request.
