# Supplementary material for: Effect of beneficial microbes applications on nutritional profiles of organic tomatoes revealed by LC‐MS‐qTOF metabolomics
Source: J Sci Food Agric. 2025 Nov 14;106(4):2091–9. doi: 10.1002/jsfa.70316 (PMC12872249; doi:10.1002/jsfa.70316)
Supplement: Supplementary file 1 — Figure S1. Layout of the experimental design. Tomato plants were treated with T. harzianum M10 (M10), T. afroharzianum T22 (T22), Streptomyces microflavus AtB‐42 (S) and two microbial consortia (S_M10 and S_T22). CTRL refers to untreated plants. Figure S2. Validation of the PLS‐DA model (positive ionization mode dataset). Left: Cross‐validation plot (5‐fold CV): performance metrics for the PLS‐DA model assessed across one to five components. The plot illustrates the model's goodness of fit (R 2), predictive ability (Q 2) and classification accuracy. Right: Permutation test (100 iterations): histogram illustrating the distribution of test statistics from 100 random permutations of the class labels (null distribution). Figure S3. Validation of the PLS‐DA model (negative ionization mode dataset). Left: Cross‐validation plot (5‐Fold CV): performance metrics for the PLS‐DA model assessed across one to five components. The plot illustrates the model's goodness of fit (R 2), predictive ability (Q 2) and classification accuracy. Right: Permutation test (100 iterations): histogram illustrating the distribution of test statistics from 100 random permutations of the class labels (null distribution). Figure S4. Important features (unidentified and identified) of tomato berries extracts, identified by partial least square discriminant analysis. Plants were treated with single strain or microbial consortia inoculants (T. harzianum M10, T. afroharzianum T22 and S. microflavus AtB‐42). Data were obtained by LC‐MS‐qTOF analysis. The first 15 features are reported from the highest to the lowest VIP value. The colored boxes on the right indicate the relative abundances of the corresponding metabolite in each group. Left: PLS‐DA scores plot in electrospray ionization in positive mode (ESI+). Right: PLS‐DA scores plot in electrospray ionization in positive mode (ESI−). Table S1. Important features (unidentified and identified) of tomato berries extracts, obtained by partial least square [file JSFA-106-2091-s001.docx]

**Supplementary data**

**Effect of beneficial microbes applications on** **nutritional profiles of organic tomatoes revealed by LC-MS qTOF metabolomics**

**Daria Lotito^1†^, Alessia Staropoli^1,2†^*, Maria Isabella Prigigallo^3^, Giuseppina Iacomino^4^, Claudio Gigliotti^2^, Giovanni Bubici^3^, Sergio Bolletti-Censi^5^, Matteo Lorito^4^, Francesco Vinale^1,2^**

^1^Department of Veterinary Medicine and Animal Productions, University of Naples Federico II, 80137 Naples, Italy; daria.lotito@unina.it (D.L.) frvinale@unina.it (F.V.)

^2^Institute for Sustainable Plant Protection, National Research Council, 80055 Portici, Italy; claudio.giglio@live.it (C.G.).

^3^Institute for Sustainable Plant Protection, National Research Council, 70126 Bari, Italy; mariaisabella.prigigallo@ipsp.cnr.it (M.I.P); giovanni.bubici@ipsp.cnr.it (G.B.)

^4^Department of Agricultural Sciences, University of Naples Federico II, 80055 Portici, Italy; giuseppina.iacomino@unina.it (G.I.); lorito@unina.it (M.L.)

^5^Cosvitec Società Consortile a Responsabilità Limitata, 80142 Naples, Italy; sergiobolletti@cosvitec.eu (S.B.C).

^†^These authors contributed equally to this work.

* Correspondence: alessia.staropoli@unina.it


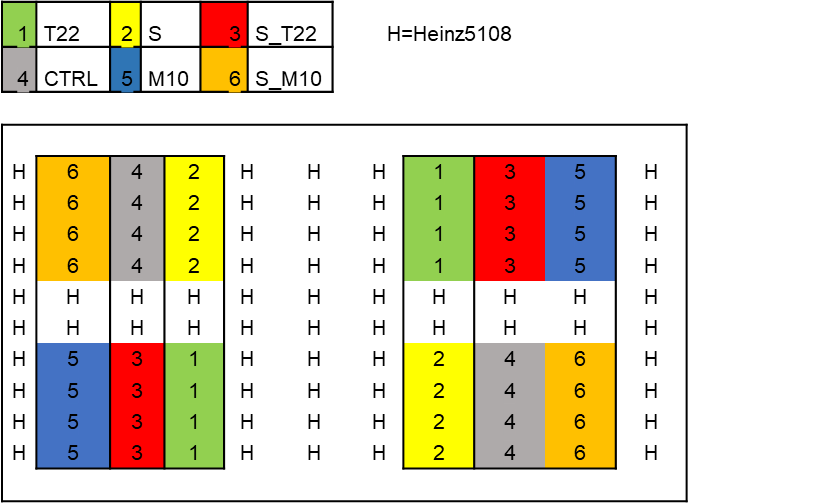


**Figure S1.** Layout of the experimental design. Tomato plants were treated with *T. harzianum* M10 (M10), *T. afroharzianum* T22 (T22), *Streptomyces microflavus* AtB-42 (S) and two microbial consortia (S_M10 and S_T22). CTRL refers to untreated plants.


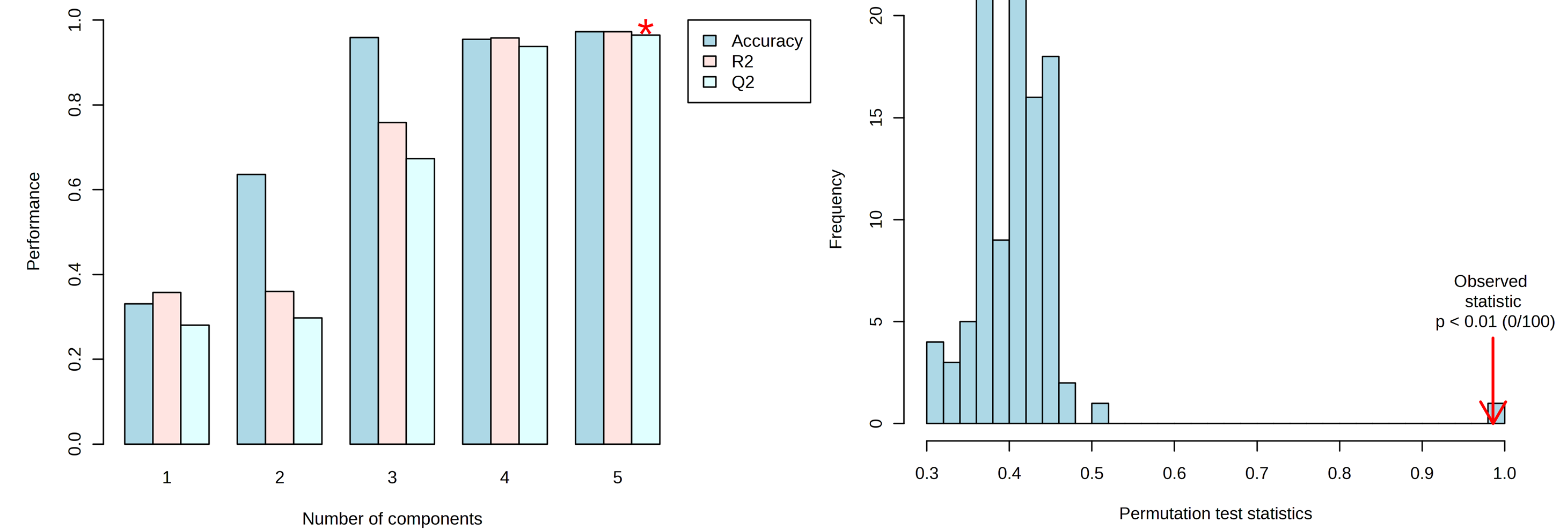


**Figure S2.** Validation of the PLS-DA Model (Positive Ionization Mode dataset). (Left) Cross-Validation Plot (5-Fold CV): Performance metrics for the PLS-DA model assessed across one to five components. The plot illustrates the model's goodness of fit (R^2^), predictive ability (Q^2^), and classification accuracy. (Right) Permutation Test (100 Iterations): Histogram illustrating the distribution of test statistics from 100 random permutations of the class labels (null distribution).


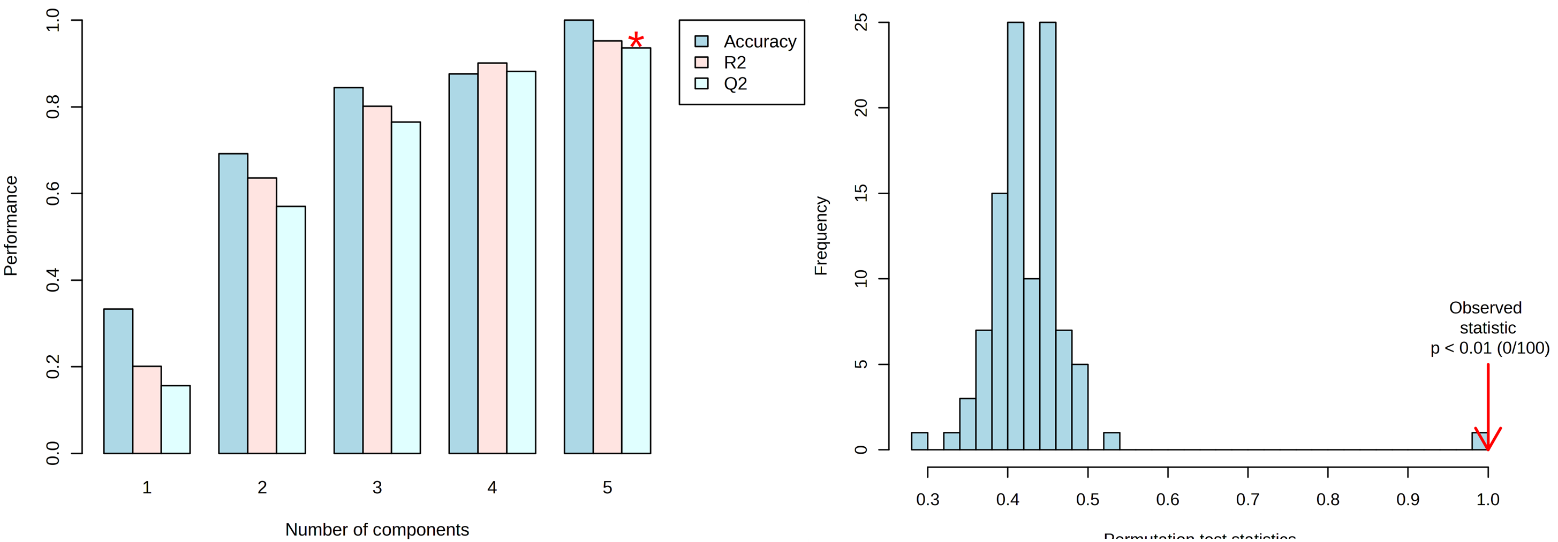


**Figure S3.** Validation of the PLS-DA Model (Negative Ionization Mode dataset). (Left) Cross-Validation Plot (5-Fold CV): Performance metrics for the PLS-DA model assessed across one to five components. The plot illustrates the model's goodness of fit (R^2^), predictive ability (Q^2^), and classification accuracy. (Right) Permutation Test (100 Iterations): Histogram illustrating the distribution of test statistics from 100 random permutations of the class labels (null distribution).

**
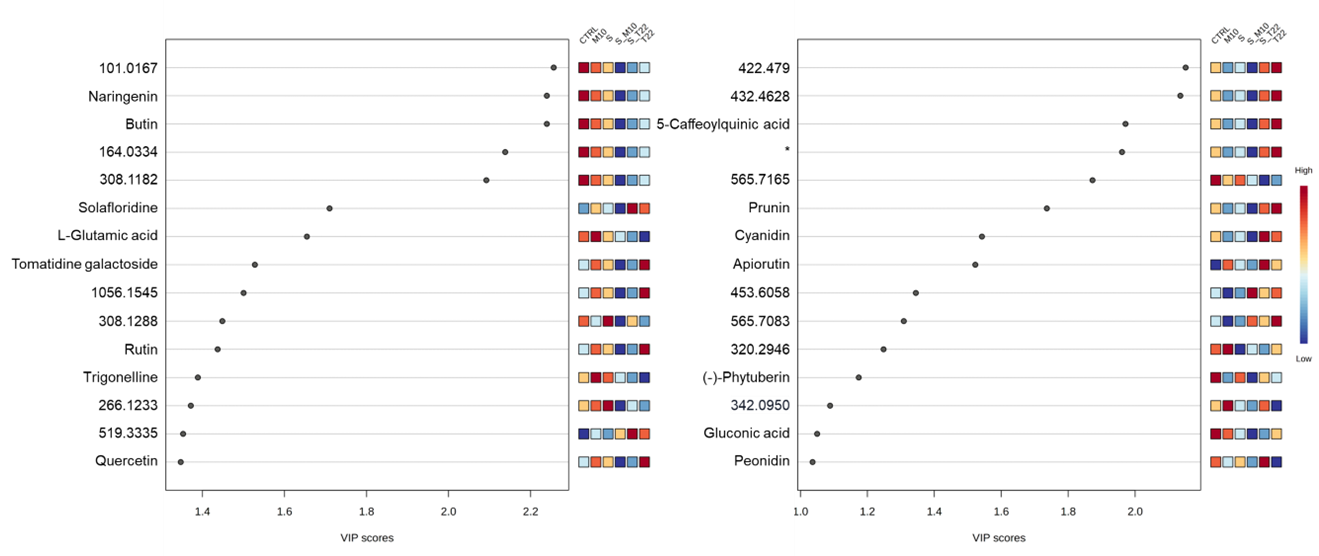
**

**Figure S4.** Important features (unidentified and identified) of tomato berries extracts, identified by Partial Least Square Discriminant Analysis. Plants were treated with single strain or microbial consortia inoculants (*T. harzianum* M10, *T. afroharzianum* T22, *S. microflavus* AtB-42). Data were obtained by LC-MS qTOF analysis. The first 15 features are reported from the highest to the lowest VIP value. The colored boxes on the right indicate the relative abundances of the corresponding metabolite in each group. Left: PLS-DA scores plot in ElectroSpray Ionization in positive mode (ESI^+^). Right: PLS-DA scores plot in ElectroSpray Ionization in positive mode (ESI^-^).

**Table S1.** Important features (unidentified and identified) of tomato berries extracts, obtained by Partial Least Square Discriminant Analysis. Plants were treated with single strain or microbial consortia inoculants (*T. harzianum* M10, *T. afroharzianum* T22, *S. microflavus* AtB-42). Data were obtained by LC-MS qTOF analysis with ElectroSpray Ionization in positive (ESI^+^) and negative (ESI^-^) mode. Variable importance in projection values are calculated for each component of PLS-DA.

| Important feature | Comp1 | Comp2 | Comp3 | Comp4 | Comp5 | Comp6 | Comp7 | Comp8 |
| --- | --- | --- | --- | --- | --- | --- | --- | --- |
| **ESI+** | | | | | | | | |
| 101.0167 | 2.256 | 2.160 | 2.124 | 2.108 | 2.100 | 2.097 | 2.094 | 2.093 |
| Naringenin | 2.239 | 2.132 | 2.102 | 2.083 | 2.073 | 2.069 | 2.067 | 2.063 |
| Butin | 2.138 | 2.132 | 2.102 | 2.083 | 2.073 | 2.069 | 2.067 | 2.063 |
| 164.0334 | 2.138 | 2.038 | 2.011 | 1.993 | 1.984 | 1.981 | 1.980 | 1.977 |
| 308.1182 | 2.092 | 2.002 | 1.971 | 1.954 | 1.945 | 1.941 | 1.939 | 1.936 |
| Solafloridine | 1.709 | 1.624 | 1.598 | 1.590 | 1.593 | 1.592 | 1.591 | 1.589 |
| L-Glutamic acid | 1.654 | 1.570 | 1.560 | 1.549 | 1.544 | 1.540 | 1.538 | 1.536 |
| Tomatidine galactoside | 1.527 | 1.468 | 1.444 | 1.432 | 1.428 | 1.426 | 1.427 | 1.425 |
| 1056.1545 | 1.500 | 1.451 | 1.428 | 1.416 | 1.413 | 1.411 | 1.412 | 1.410 |
| 308.1288 | 1.448 | 1.365 | 1.346 | 1.356 | 1.349 | 1.347 | 1.347 | 1.345 |
| Rutin | 1.437 | 1.421 | 1.406 | 1.394 | 1.400 | 1.397 | 1.400 | 1.398 |
| Trigonelline | 1.388 | 1.360 | 1.338 | 1.331 | 1.326 | 1.323 | 1.322 | 1.319 |
| 266.1233 | 1.371 | 1.368 | 1.346 | 1.337 | 1.331 | 1.331 | 1.330 | 1.328 |
| 519.3335 | 1.352 | 1.280 | 1.271 | 1.263 | 1.261 | 1.259 | 1.257 | 1.256 |
| Quercetin | 1.347 | 1.322 | 1.300 | 1.290 | 1.286 | 1.286 | 1.285 | 1.285 |
| 165.0425 | 1.151 | 1.087 | 1.080 | 1.076 | 1.113 | 1.117 | 1.116 | 1.114 |
| 129.0406 | 1.091 | 1.141 | 1.129 | 1.120 | 1.115 | 1.112 | 1.111 | 1.109 |
| 115.0502 | 1.004 | 0.966 | 0.971 | 0.998 | 0.999 | 1.007 | 1.006 | 1.007 |
| Tomatidine | 1.004 | 0.953 | 0.938 | 0.951 | 0.993 | 0.991 | 0.990 | 0.990 |
| **ESI-** | | | | | | | | |
| 422.479 | 2.150 | 2.075 | 2.034 | 2.033 | 2.026 | 2.020 | 2.016 | 2.014 |
| 432.4628 | 2.135 | 2.058 | 2.018 | 2.017 | 2.010 | 2.004 | 2.001 | 1.999 |
| 5-Caffeoylquinic acid | 1.971 | 1.902 | 1.864 | 1.862 | 1.855 | 1.850 | 1.846 | 1.844 |
| trans-p-Ferulyl alcohol 4-O-[6-(2-methyl-3-hydroxypropionyl)] glucopyranoside | 1.960 | 1.890 | 1.853 | 1.849 | 1.845 | 1.840 | 1.836 | 1.834 |
| 565.7165 | 1.872 | 1.806 | 1.777 | 1.773 | 1.766 | 1.762 | 1.758 | 1.756 |
| Prunin | 1.736 | 1.681 | 1.648 | 1.644 | 1.641 | 1.638 | 1.634 | 1.633 |
| Cyanidin | 1.542 | 1.529 | 1.503 | 1.500 | 1.496 | 1.492 | 1.489 | 1.488 |
| Apiorutin | 1.522 | 1.496 | 1.468 | 1.464 | 1.460 | 1.456 | 1.453 | 1.452 |
| 453.6058 | 1.344 | 1.365 | 1.354 | 1.350 | 1.345 | 1.341 | 1.338 | 1.344 |
| 565.7083 | 1.308 | 1.301 | 1.276 | 1.273 | 1.269 | 1.267 | 1.265 | 1.264 |
| 320.2946 | 1.248 | 1.232 | 1.265 | 1.265 | 1.261 | 1.26 | 1.265 | 1.266 |
| (-)-Phytuberin | 1.174 | 1.149 | 1.146 | 1.148 | 1.147 | 1.144 | 1.142 | 1.140 |
| 342.095 | 1.088 | 1.094 | 1.103 | 1.100 | 1.098 | 1.113 | 1.111 | 1.112 |
| Gluconic acid | 1.049 | 1.013 | 0.994 | 1.015 | 1.014 | 1.013 | 1.012 | 1.011 |
| Peonidin | 1.036 | 1.079 | 1.057 | 1.054 | 1.052 | 1.05 | 1.047 | 1.047 |
| Kaempferol 3,7-di-O-glucoside | 1.034 | 1.077 | 1.056 | 1.053 | 1.051 | 1.048 | 1.046 | 1.045 |

**Table S2.** Significantly different molecular features (unidentified and identified) of tomato berries extracts, obtained by analysis of variance (ANOVA, p<0.05). Plants were treated with single strain or microbial consortia inoculants (*T. harzianum* M10, *T. afroharzianum* T22, *S. microflavus* AtB-42). Data were obtained by LC-MS qTOF analysis with ElectroSpray Ionization in positive (ESI^+^) and negative (ESI^-^) mode..

| Molecular feature | p |
| --- | --- |
| **ESI+** | |
| 265.2608 | 4.85E-32 |
| Rutin | 9.38E-32 |
| 101.1515 | 9.99E-32 |
| 103.1465 | 5.49E-30 |
| 218.2954 | 8.17E-30 |
| 813.9536 | 1.06E-29 |
| 1056.1546 | 1.48E-27 |
| Naringenin | 3.57E-27 |
| Butin | 3.57E-27 |
| Tomatidine galactoside | 1.33E-26 |
| 101.5539 | 1.19E-25 |
| (-)-Epicatechin | 3.50E-23 |
| 165.1299 | 1.40E-22 |
| Quercetin | 1.81E-22 |
| 317.5079 | 4.51E-22 |
| 119.1392 | 1.06E-21 |
| 308.3569 | 3.52E-21 |
| 3-Indolyllactic acid | 5.93E-21 |
| 101.1041 | 2.02E-20 |
| 308.2691 | 3.06E-20 |
| 165.1464 | 3.50E-20 |
| 129.0977 | 3.62E-20 |
| 115.1307 | 1.49E-19 |
| 135.1005 | 2.18E-19 |
| 267.2154 | 6.89E-18 |
| Tyramine | 3.38E-16 |
| Lycoperoside F | 1.08E-15 |
| 265.2443 | 1.01E-14 |
| 495.6697 | 1.96E-14 |
| 137.0932 | 9.98E-14 |
| 257.4558 | 1.20E-12 |
| 477.5153 | 1.51E-12 |
| L-Homoserine | 7.65E-12 |
| Solafloridine | 1.81E-10 |
| 119.1866 | 3.06E-10 |
| Adenine | 3.80E-10 |
| Sucrose | 5.37E-10 |
| 102.1561 | 6.52E-10 |
| 102.0689 | 7.05E-10 |
| 115.1125 | 8.65E-10 |
| Kaempferol 3,7-di-O-glucoside | 9.88E-10 |
| 626.7386 | 1.04E-09 |
| 115.1108 | 1.23E-09 |
| 165.1514 | 4.85E-09 |
| Trigonelline | 8.15E-09 |
| 23R-Acetoxytomatine | 9.32E-09 |
| Tomatidine | 4.79E-08 |
| 267.1557 | 8.39E-08 |
| L-Glutamic acid | 1.89E-07 |
| 129.0943 | 3.36E-07 |
| 103.1631 | 4.58E-07 |
| 159.1684 | 5.30E-07 |
| Adenosine | 1.06E-06 |
| Indole-3-ethanol | 1.17E-06 |
| 477.6113 | 1.77E-06 |
| 1056.1711 | 2.41E-06 |
| delta-Tomatine | 6.90E-06 |
| 495.5655 | 9.23E-06 |
| 519.6995 | 1.85E-05 |
| Tomatine | 0.000106 |
| 265.1746 | 0.000125 |
| Linamarin | 0.000137 |
| 519.5870 | 0.000193 |
| Naringenin | 0.002757 |
| **ESI-** | |
| Apiorutin | 1.56E-30 |
| Caffeic acid 3-glucoside | 2.44E-30 |
| 422.479014 | 2.11E-26 |
| Quercetin 3-galactoside-7-rhamnoside | 6.28E-26 |
| Kaempferol 3,7-di-O-glucoside | 1.31E-25 |
| 611.468721 | 5.41E-25 |
| 432.462871 | 1.47E-24 |
| 3-[4-(beta-D-Glucopyranosyloxy)-3-hydroxyphenyl]-2-propenoic acid | 1.02E-23 |
| 380.459383 | 9.44E-21 |
| L-Glutamine | 3.48E-19 |
| 426.415227 | 8.80E-19 |
| 273.241419 | 1.33E-18 |
| trans-p-Ferulyl alcohol 4-O-[6-(2-methyl-3-hydroxypropionyl)] glucopyranoside | 7.09E-18 |
| 188.22464 | 7.33E-18 |
| 5-Caffeoylquinic acid | 7.99E-18 |
| 356.437911 | 1.29E-17 |
| Peonidin | 7.95E-17 |
| 272.323972 | 1.00E-15 |
| (+)-Gallocatechin | 1.61E-15 |
| 453.605856 | 1.65E-15 |
| 112.083657 | 3.07E-15 |
| Prunin | 1.06E-14 |
| 192.132101 | 1.70E-12 |
| Cyanidin | 7.09E-12 |
| 5-Caffeoylquinic acid | 1.24E-11 |
| Quinic acid | 3.11E-11 |
| 453.622385 | 3.89E-11 |
| Delphin | 4.51E-11 |
| 147.137835 | 1.09E-10 |
| Mesquitol-4alpha-ol 8-methyl ether | 1.35E-10 |
| 347.301243 | 4.37E-10 |
| 1-Caffeoyl-beta-D-glucose | 5.82E-10 |
| 103.119963 | 6.62E-10 |
| 477.603071 | 2.28E-09 |
| (-)-Phytuberin | 3.70E-09 |
| 565.716554 | 3.72E-09 |
| Galactonic acid | 5.13E-09 |
| L-Tyrosine | 3.77E-08 |
| 406.236613 | 7.41E-07 |
| 565.708351 | 1.65E-06 |
| L-Homoserine | 1.73E-06 |
| 477.611275 | 2.98E-06 |
| FEMA 3677 | 6.09E-06 |
| 206.150393 | 9.41E-06 |
| 246.260803 | 0.000136 |
| Gluconic acid | 0.00025 |
| 541.695082 | 0.001072 |
| L-Asparagine | 0.002983 |
| 133.111217 | 0.005678 |

**Table S3.** Absolute Fold Change values for a selected group of differentially accumulated metabolites in tomato berries. Metabolites were initially selected based on Partial Least Squares-Discriminant Analysis (PLS-DA) and analysis of variance (ANOVA). Fold Change values >1 indicate up-regulation (enrichment), and values <1 indicate down-regulation (depletion). Data were obtained by LC-MS Q-TOF analysis in both positive and negative ionization mode. Plants were treated with single strain or microbial consortia inoculants (*T. harzianum* M10, *T. afroharzianum* T22, *S. microflavus* AtB-42).

| **Compound** | **Fold Change (absolute)** | | | | | | | | |
| --- | --- | --- | --- | --- | --- | --- | --- | --- | --- |
|  | **S vs Ctrl** | **M10 vs Ctrl** | **T22 vs Ctrl** | **S_M10 vs Ctrl** | **S_T22 vs Ctrl** | **S_M10 vs S** | **S_M10 vs M10** | **S_T22 vs S** | **S_T22 vs T22** |
| (-)-Phytuberin | - | - | - | 0.34752 | 0.3232 | 0.40214 | - | 0.374 | 0.36201 |
| 5-Caffeoylquinic acid | 0.11937 | 0.11937 | - | - | 0.11937 | 8.6939 | 8.6939 | - | 0.10031 |
| Apiorutin | 14.633 | 16.535 | 26.548 | 11.71 | 21.85 | - | - | - | - |
| Butin | 0.1518 | 0.1518 | 0.1518 | 0.1518 | 0.1518 | - | - | - | - |
| Cyanidin | - | - | 9.9265 | - | 4.8954 | - | - | 4.8954 | 0.49317 |
| Gluconic acid | 0.32582 | - | - | 0.034795 | 0.14299 | 0.10679 | 0.032942 | - | 0.15085 |
| Kaempferol 3,7-di-O-glucoside | - | - | - | - | -- | - | - | - | - |
| L-Glutamic acid | 0.38534 | 0.40455 | 0.35548 | - | - | 2.6289 | 2.504 | 2.2484 | 2.4373 |
| Naringenin | - | - | - | 2.628 | - | 15.368 | 24.189 | - | - |
| Peonidin | 6.3814 | - | 8.8835 | - | - | 0.15671 | - | 0.15671 | 0.11257 |
| Prunin | - | - | 19.265 | - | - | - | - | - | 0.051908 |
| Quercetin | - | - | 7.125 | - | - | - | - | - | 0.14035 |
| Rutin | - | - | 25.333 | - | - | - | - | - | - |
| Solafloridine | - | - | 31.584 | - | 34.254 | - | - | 34.254 | - |
| Tomatidine | 0.076227 | 0.07622 | 2.733 | - | - | 13.554 | 13.554 | 15.674 | - |
| Tomatidine galactoside | - | - | 10.61 | - | - | - | - | - | 0.094253 |
| trans-p-Ferulylalcohol 4-O-[6-(2-methyl-3-hydroxypropionyl)] glucopyranoside | - | - | 8.3536 | - | - | - | - | - | 0.11971 |
| Trigonelline | - | - | 0.49912 | - | - | 2.093 | 0.15068 | - | - |

The dash (-) indicates that the metabolite did not meet the Fold Change cut-off for that comparison.
